# Reimagining the relationship between Gondwanan forests and Aboriginal land management in Australia's “Wet Tropics”

**DOI:** 10.1016/j.isci.2021.102190

**Published:** 2021-02-16

**Authors:** Patrick Roberts, Alice Buhrich, Victor Caetano-Andrade, Richard Cosgrove, Andrew Fairbairn, S. Anna Florin, Nils Vanwezer, Nicole Boivin, Barry Hunter, Desley Mosquito, Gerry Turpin, Åsa Ferrier

**Affiliations:** 1 Department of Archaeology, Max Planck Institute for the Science of Human History, Germany; 2 School of Social Science, The University of Queensland, Brisbane, Australia; 3 College of Arts, Society and Education, James Cook University, Cairns, Australia; 4 Department of Archaeology and History, La Trobe University, Melbourne, Australia; 5 Australian Research Council Centre of Excellence for Australian Biodiversity and Heritage, University of Wollongong, Wollongong, Australia; 6 Djabugay Aboriginal Corporation, Kuranda, Australia; 7 Wabubadda Aboriginal Corporation RNTBC, Jirrbal Aboriginal People, Atherton, Australia; 8 Tropical Indigenous Ethnobotany Centre, Australian Tropical Herbarium, James Cook University, McGregor Road, Smithfield, QLD 4879, Australia; 9 Queensland Herbarium, Department of Environment and Science, Mount Coot-tha Botanical Gardens, Mount Cooth-tha Road, Toowong, QLD 4066, Australia

**Keywords:** Environmental Science, Environmental Monitoring, Nature Conservation, Environmental Resource, Biological Sciences, Plant Biology, Botany, Plant Ecology, Ethnobotany, Agricultural Science

## Abstract

The “Wet Tropics” of Australia host a unique variety of plant lineages that trace their origins to the super-continent of Gondwanaland. While these “ancient” evolutionary records are rightly emphasized in current management of the region, multidisciplinary research and lobbying by Rainforest Aboriginal Peoples have also demonstrated the significance of the cultural heritage of the “Wet Tropics.” Here, we evaluate the existing archeological, paleoenvironmental, and historical evidence to demonstrate the diverse ways in which these forests are globally significant, not only for their ecological heritage but also for their preservation of traces of millennia of anthropogenic activities, including active burning and food tree manipulation. We argue that detailed paleoecological, ethnobotanical, and archeological studies, working within the framework of growing national and world heritage initiatives and active application of traditional knowledge, offer the best opportunities for sustainable management of these unique environments in the face of increasingly catastrophic climate change and bushfires.

## Graphical abstract

**Figure:**
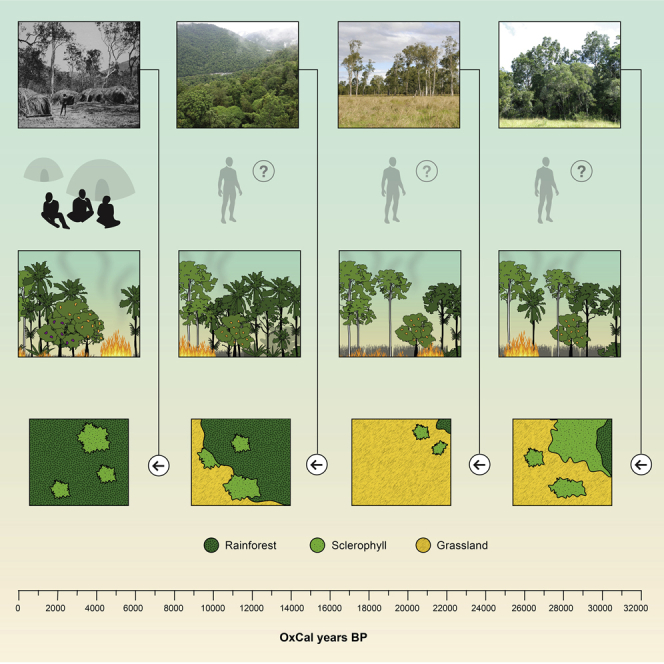

## Introduction

**Figure 1:**
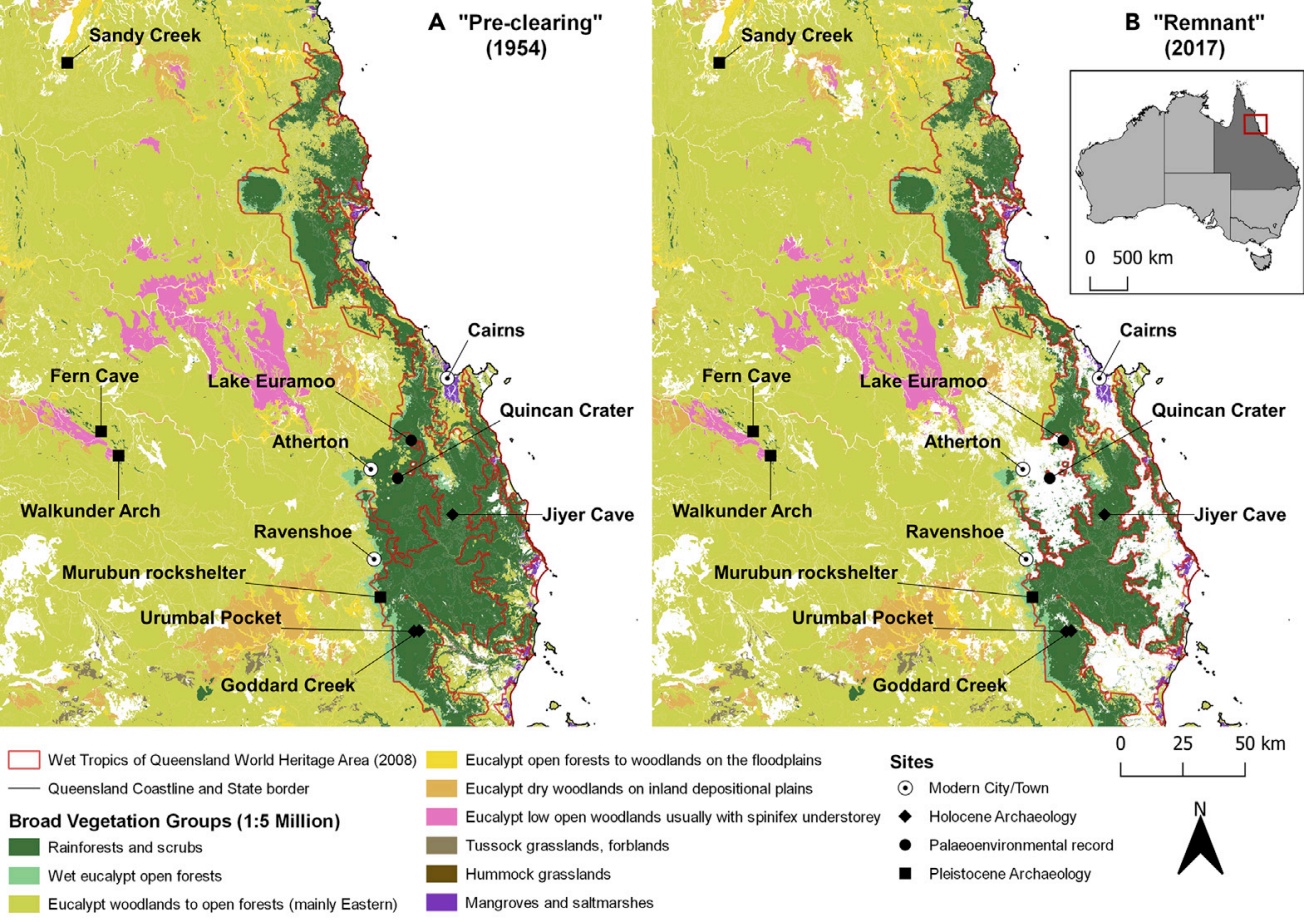
Map of the mentioned archeological and paleoenvironmental sites and their location in relation to the Wet Tropics Bioregion of Queensland, Australia Changes in general vegetation groups (Neldner et al., 2019) within, and in the vicinity of, the Wet Tropics Bioregion of Queensland, Australia (Wet Tropics Management Authority, 2013) are shown: (A) before the clearance of major forested areas in 1954 and (B) their remnant extents in 2017.

The Wet Tropics Bioregion and World Heritage Area of Queensland, north-eastern Australia (Figure 1), is argued to preserve “*the oldest surviving rainforest in the world*” (Wet Tropics Management Authority, 2015). Before the 20^th^ century, it was thought that these tropical rainforest environments were “alien” and “invasive,” representing an incursion of Asiatic species. However, the last half a century of research has revealed that the Wet Tropics host uniquely high concentrations of all plant groups, including angiosperm and gymnosperm species, with evolutionary histories tracing back to Gondwanaland (Webb and Tracey, 1994; Sanderson, 2008), containing 19 of 26 near basal families of dicotyledonous plants and 626 species endemic to Australia (Metcalfe and Ford, 2009; Wet Tropics Management Authority, 2016). Beyond the rainforest, more open, drier, and varied sclerophyll forests host a further variety of endemic Australian plant species (Tracey, 1982). Although the Wet Tropics covers only 0.12% of Australia, it also contains approximately 45% of all Australian terrestrial vertebrate species, a quarter of which have special conservation value (Wet Tropics Management Authority, 2016). Unsurprisingly, these findings from decades of rigorous ecological and paleoecological research have meant that the World Heritage listing of the “Wet Tropics” emphasizes its hosting of “*relicts of the great Gondwanan forest that covered Australia … 50–100 million years ago*” (UNESCO, 2020) and its representation of ecological processes that have fundamentally shaped the biota of Australia (UNESCO, 2020). The declaration of the Wet Tropics of Queensland as an 894,420-hectare World Heritage Area in 1988 has resulted in the arrival of 5,000,000 tourists and $ 5.2 billion (AUS) annually to the region (Wet Tropics Management Authority, 2015).

Not currently mentioned as part of its international UNESCO World Heritage listing, however, is the cultural significance of the “Wet Tropics,” and, traditionally, management policies have restricted Rainforest Aboriginal Peoples' access to its forest environments, favoring botanical novelty and evolutionary trajectories over human history. Combined with a loss of rainforest to agriculture and urban development, these restrictions have resulted in significant and rapid changes to the vegetation cover of the region (Stanton et al., 2014a, 2014b). This is despite the fact that the Wet Tropics is the current home of at least 20 Aboriginal groups, 120 clans, and 8 languages (although this number is dynamic), whose ancestors have occupied the landscape for millennia (Pannell, 2005; Cosgrove et al., 2007; Ferrier, 2015) and made use of a number of the ecologically significant plants that the region has to offer (Tuechler et al., 2016; Wet Tropics Management Authority, 2020b). As shown elsewhere in Australia, use of European census and ethnographic observation data has often obscured the potential size of pre-colonial populations that existed before introduced disease, genocide, and relocation (Pascoe, 2018). Moreover, in other parts of Australia, researchers and Aboriginal Traditional Owners have increasingly drawn upon archeological, ethnohistoric, and paleoecological datasets to argue that large communities actively managed a diversity of landscapes before the onset of Australia's early colonial period (1788–1850) (Lourandos and David, 1998; Hill and Gribbs, 2000; Gammage, 2012; Pascoe, 2018). This included plant cultivation, including yams (*Dioscorea hastifolia*) and millet (*Panicum decompositum*) (Hallam, 1989; Pascoe, 2018); active burning to maintain mosaics of forest, grassland, and sedge environments to promote populations of medium-sized mammals (Jones, 1979; Jurskis, 2009; Gammage, 2012; Fletcher et al., 2020); water management, such as damming to maintain wetland vegetation communities (Barber and Jackson, 2015); the development of complex trapping structures to obtain freshwater resources (Hallam, 1989; Builth et al., 2008); and the formation of seasonally occupied settlements (Ferrier 2015; Pascoe, 2018).

In the “Wet Tropics,” campaigning by Rainforest Aboriginal Peoples, and their supporters, has started to shift perspectives and practices, in a similar way to recent research elsewhere in Australia (Pascoe, 2018; Fletcher et al., 2020), as well as in the Amazon Basin (Levis et al., 2017), which has encouraged a transition away from a focus on purely “natural” forests toward an understanding of the role Indigenous populations have played in shaping and managing the biodiversity that exists today (Australian Government Department of Agriculture, Water and the Environment, 2021a). The most recent Australian National Heritage listing for the Wet Tropics of 2012 includes a statement of cultural significance:

“*The Aboriginal Rainforest People of the Wet Tropics of Queensland have lived continuously in the rainforest environment for at least 5,000 years and this is the only place in Australia where Aboriginal people have permanently inhabited a tropical rainforest environment*.” (Australian Government Department of Agriculture, Water and the Environment, 2021b).

Ethnographic and historical ecological insights have also documented the significance of the cultivation of patches of cycads and fruit trees and use of fire to maintain “healthy country” among the Yalanji in the northern Wet Tropics (Hill and Gribbs, 2000; Hill and Baird, 2003; Hill et al., 2004). The Wet Tropics Management Authority now requires two of the seven Board of Director positions to be held by Aboriginal people and is increasingly raising the profile of the cultural values of the region. Rainforest Aboriginal Peoples, who have carefully documented their knowledge about being custodians and knowledge-holders for the region's forests (Hill et al., 2011), are also being increasingly administratively supported in the setting up of ranger groups; the application of traditional management practices, including cultural burning, to forest landscapes; the development of a consultative Traditional Owner Leadership Group; and the creation of Indigenous Protected Areas (Wet Tropics Aboriginal Cultural and Natural Plan Project Team 2005; National Environmental Research Program, 2014; Garnett et al., 2017; Wet Tropics Management Authority, 2020b; National Indigenous Australians Agency, 2021). Rainforest Aboriginal People have been campaigning for the recognition of the role of Aboriginal land managers in shaping and managing the Wet Tropics since the World Heritage Area was declared, when Rainforest Aboriginal groups produced a review of Aboriginal interests in the world heritage nomination, provocatively titled “Which way our cultural survival”? (Review Steering Committee, 1998). A campaign led by Rainforest Aboriginal People continues to push for re-listing of the “Wet Tropics” as a UNESCO site of both natural and cultural heritage (Cultural Values Project Steering Committee, 2016). However, support remains somewhat patchy for the different Aboriginal groups across the region, and the process of altering the region's world heritage status is not yet finalized.

In this article, we seek to draw upon a variety of multidisciplinary datasets that are yet to be compiled in a peer-reviewed publication, to support growing movements looking to re-cast the forests of the Wet Tropics as cultural landscapes as well as purely “primitive” Gondwanan remnants. We suggest that previous estimations of pre-colonial Aboriginal populations are gross underestimates and that subsequent European activities have hidden the full scale of prehistoric landscape management behind a western environmental determinism, long-term colonial rationales for dispossession, and a lack of acceptance of the scale of ensuing atrocities on Indigenous demography and land management (Pascoe, 2018). We review evidence for human presence from the Late Pleistocene to European invasion (~45,000 years ago to 1860 AD) before highlighting evidence of deliberate burning to create and preserve productive “pockets” composed of sclerophyll forest and grassy vegetation within rainforest environments, the potential tending and planting of important food plants, and the management of wild fauna. We suggest that these insights include the “Wet Tropics” within a growing global recognition that pre-industrial human management of, and impact on, tropical forests, although once considered limited (e.g., Meggers, 1971; Bailey and Headland, 1991), was in fact significant, leaving major legacies for the 21^st^ century environments and ecological dynamics, and making them “hotspots” of “anthropocene” processes (Roberts et al., 2017a, 2017b, 2018; Levis et al., 2017). We argue that these datasets, recent catastrophic bushfire events (Gergis, 2019), and climate change underpin the growing move in conservation policy within the Wet Tropics toward promoting close dialog between archeological and paleoecological research, Aboriginal cultural values and knowledge, ecological and conservation priorities, and land management. In this way, it is possible to explore long-term landscape and biota change, the application of “On Country” management strategies, and the re-classification of the Wet Tropics to recognize the international significance of its multi-faceted heritage.

### A changing human presence in the Wet Tropics

The Wet Tropics is home to a diversity of forest types that have seen significant temporal fluctuations in their distribution in the past (Kershaw et al., 2007). Tracey and Webb (1975) and Tracey (1982) have developed a detailed structural classification for rainforest and related vegetation types in the Wet Tropics. Thirteen sub-formations of rainforest, as well as 10 types of rainforest with emergent sclerophylls, have been identified. Three *main* types of tropical forest, primarily dictated by geology and precipitation, exist in the Wet Tropics (Figure 1) (Wet Tropics Management Authority, 2013; Neldner et al., 2019): complex mesophyll and notophyll vine forests occur in granite soils in wet, lowland (mainly <300 m asl) areas, along river valleys and in basaltic soils in the Atherton Tableland region (averaging 400–1,280 m asl); wet sclerophyll forest types dominated by eucalypts occur in slightly drier areas; and dry sclerophyll eucalypt forest and woodland (including Acacia forests) exist in arid areas (Harrington and Sanderson, 1994; Van der Wal et al., 2009; Haberle et al., 2010; Stanton et al., 2014a, 2014b) (Figure 1). Rainforest types are commonly characterized ecologically as being fire-resistant and damaged by frequent burning events (Harrington and Sanderson, 1994; Bowman and Cook, 2001), in contrast to the wet and dry sclerophyll forests and woodlands, which are considered to be actively adapted to natural and anthropogenic fires (Bowman and Cook, 2001; Haberle et al., 2010). Significantly, the proportions of these forest types have fluctuated through time, with sclerophyll woodland more expansive during the Late Pleistocene and rainforest and wet sclerophyll proliferation occurring in the Early and Late Holocene (Van der Wal et al., 2009; Haberle et al., 2010). Since the 1980s, and the management of the Wet Tropics as a World Heritage Area, there has been a significant encroachment of sclerophyll forest types by rainforest (Kemp et al., 2007; Stanton et al., 2014a, 2014b).

The earliest suggested evidence for human presence in what is now known as the Wet Tropics Bioregion (which at that time was covered by extensive open sclerophyll forest) comes in the form of spikes in biomass burning *c*. 45,000 years ago in paleoenvironmental records from the Atherton Tablelands (Kershaw, 1986; Turney et al., 2001; Hilbert et al., 2007). However, extensive surveys have failed to find archeological evidence dating to this period (Cosgrove et al., 2007). There is archeological evidence *c*. 35,000 years ago at Walkunder Arch, Fern Cave, Sandy Creek, and Ngarrabullgan, all within 100 km of the Wet Tropics (Campbell, 1984; David and Chant, 1995), and Pleistocene occupation was also recorded at Murubun rockshelter on the western edge of the Atherton Tablelands *c*. 31,000 years ago (Cosgrove et al., 2007) (Figure 1). Within the Wet Tropics, the first clear evidence for human occupation, and indeed use of rainforest or sclerophyll resources, only occurs *c*. 8,000 cal. years BP at the site of Urumbal Pocket (Cosgrove et al., 2007). Human presence remains sporadic until 2,500–1,500 cal. years BP when increases in artifact discard rates at Urumbal Pocket, and at several other sites across the Wet Tropics, become apparent. As such, the tempo of Aboriginal occupation within the Wet Tropics is somewhat unique within Australia more broadly (Cosgrove, 1996). Elsewhere, following arrival *c*. 65,000–55,000 years ago, populations moved into a diverse array of environments by at least 47,000 years ago (McDonald et al., 2018). In the Wet Tropics, clear human occupation is evident only in the Holocene.

This “late” signature may in part be due to the fact that much of Australia's archeological record is in the form of undated lithic scatters that are difficult to discern in the densely vegetated environments of the Wet Tropics (Anderson and Robins, 1988; Cosgrove et al., 2007). The strongest available evidence for earlier habitation comes from oral histories of the Traditional Owners, who describe the creation of volcanic crater lakes on the Atherton Tablelands that are known to have formed 13,000 years ago (Dixon, 1983; Pannell, 2008), implying human presence in the region in the Late Pleistocene. Meanwhile, on the coast near Cairns, oral tradition among the Gunggandji people recalls places such as *Muduaa* “the place of the pencil pines” being drowned by sea level rise in the Early Holocene (Dixon, 1983). From around 2,000 years ago, increasingly permanent human occupation becomes clearly visible across the Wet Tropics (Cosgrove et al., 2007), as has now been acknowledged in its Australian National Heritage listing (Australian Government Department of Agriculture, Water and the Environment, 2021a; 2021b). Nevertheless, there has thus far been relatively little discussion of the scale of this occupation. Commonly cited estimates of the pre-contact Aboriginal population numbers in the region, based on European census data and ethnographic analysis, lie around 5,500 people (Harris, 1978; Tracey, 1982), a number that perhaps suggests limited pre-colonial impact on the environments of the Wet Tropics. This is particularly the case given assumptions that the rainforests of the Wet Tropics would have been broadly unattractive to human settlement, a perspective also used to explain a relatively late occupation of the region, with toxic nuts and tubers, and sparse, difficult-to-catch fauna only becoming desirable in the Mid-Holocene when specialized adaptations to the attainment and processing of these foods arose in response to climate change (Cosgrove, 1996; Cosgrove et al., 2007).

**Figure 2:**
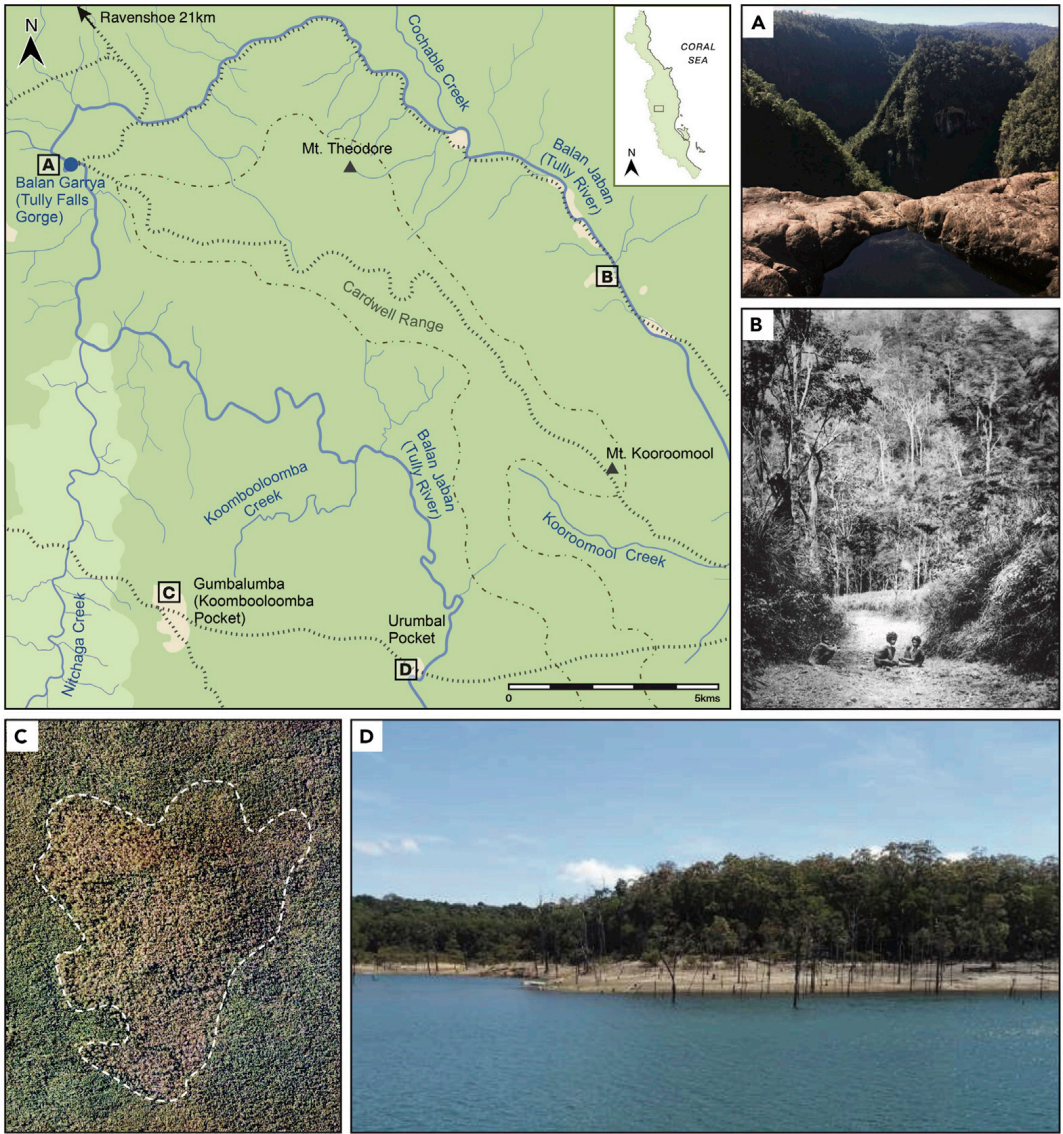
Map of Aboriginal walking tracks and major cultural landmarks in the vicinity of Koombooloomba and Urumbal Pockets Shown in terms of the area's location within the Wet Tropics Bioregion and redrawn from Queensland Tully, Sheet No74, Zone 7, 2093 Tully 1 Inch Series, Surveyed by 5 Aust Fd Svy Coy AIF, Detail from Air Photos Dec. 1943, Brisbane. (A) View of Balan Garrya (Tully Falls Gorge); (B) photograph of Aboriginal children on a maintained walking track in the Wet Tropics. Source: Courtesy John Oxley Library, Brisbane (late 1800-early 1900s); (C) aerial image of Gumbalumba (Koombooloomba) Pocket showing the clear impact of past Aboriginal fire management still visible within modern rainforest vegetation. Source: Creative Commons Attribution 4.0 license. State of Queensland. (D) View of Urumbal Pocket from Balan Jaban (Tully River).

Nonetheless, three main lines of emerging archeological, historical, and ethnographic evidence hint that the region may have been more densely populated than has often been assumed. First, the surface finds of many thousands of stone axes, grinding stones, flaked stone artifacts, and other implements, as well as a high density of documented Aboriginal camping and ceremonial grounds (Horsfall, 1996), imply much more widespread occupation of the region in the past. Second, colonial archives written at the time of European contact also imply much more widespread occupation of the Wet Tropics. For example, during the first inland expedition to Cape York, expedition leader Edmund Kennedy described the Wet Tropics landscape as “a patchwork made up of open forest pockets, rainforest and tracks” (Beale, 1977) (Figure 2), and in 1876 gold prospector James Mulligan described large clusters of huts located in open eucalypt pockets on the fringe of the rainforest on the Atherton Tablelands, which he referred to as townships:

*“There are roads off the main track to each of their townships, which consist of well-thatched huts, big enough to hold five or six people*. *We counted eleven townships since we arrived”*. (Mulligan, 1876)

Recollections by Europeans also include descriptions of thousands of Aboriginal people meeting for ceremonial gatherings (e.g., Morrill, 2006), suggesting a much higher pre-contact Aboriginal population in the region than prevailing estimates. An abundance of historical documents and oral history testimonies describe the forced relocation of Rainforest Aboriginal People to stations and missions, and the genocide of hundreds, if not thousands, of Aboriginal people from the region (e.g., Loos, 1982), likely leading to a bias in pre-contact demographic estimations. Third, Rainforest Aboriginal Peoples (Aboriginal Australian people originating from the rainforest regions of Far North Queensland), in part based on oral histories passed down from Aboriginal Elders (Pannell, 2008; Nunn and Reid, 2016; Rossetto et al., 2017), have emphasized the fact that the environments of this region are a product of generations of Aboriginal land management. Future archeological and ethnohistorical work may therefore reveal an even greater extent of pre-colonial human occupation across the Wet Tropics, as has been demonstrated elsewhere in the tropical world (Heckenberger et al., 2003; Bush et al., 2015; Koch et al., 2019).

## A dynamic relationship between humans and tropical forests in Australia

The aforementioned evidence provides a context for the tempo of *occupation* of the Wet Tropics as well as the extent of settlement at the time of European contact. There is also clear evidence that these Aboriginal societies significantly used and *manipulated* the environments of the Wet Tropics with lasting legacies. Perhaps most clear in this regard is the long-term evidence for the maintenance of environmental boundaries in the Wet Tropics through burning (e.g., Hill and Baird, 2003; Moss et al., 2012). Ethnographic records across Australia have long demonstrated that frequent, low-intensity burning, so-called fire-stick farming (Jones, 1979), was utilized by Aboriginal communities to alter plant and animal communities, increase biodiversity, reduce hazards, control weeds, and facilitate hunting (Jones, 1979; Jurskis, 2009; Gammage, 2012). Fletcher et al. (2020) have recently demonstrated how the forced cessation of Aboriginal burning following British invasion in Tasmania has led to the gradual replacement of grassland by cool temperate rainforest. Although the probability of Aboriginal burning of the fire-prone sclerophyll forests of the Wet Tropics has been considered high, traditionally, fire activity in rainforests is thought to have been rare due to high soil moisture, where fires started by lightning are extinguished by the following high rainfall (Stocker and Mott 1981; Metcalfe and Ford 2008:127). Yet, charcoal identified from two rainforest species *Halfordia* sp. and *Pouteria* sp. has been dated to 400–200 years ago in soils excavated under what was previously assumed to be “natural” modern rainforest (Cosgrove, 2005). Graham (2006:82) also recovered charcoal from six rainforest locations dating to between c. 780 and c. 7,790 years ago. The presence of charcoal in rainforest soils therefore suggests these fires were likely caused by human ignition, which, in turn, indicates a long history of Aboriginal burning to manage rainforest environments, including the opening of patches of forest for ceremonial grounds and the clearing of walking tracks and camp sites through traditional burning methods such as the placing of hot coals at the base of lawyer vines (Australian Government Department of Agriculture, Water and the Environment, 2021a).

Indeed, available archeological and paleoecological data suggest that Aboriginal burning was likely a key force in the Wet Tropics pre-contact. Although claims of deliberate human burning *c*. 45,000–30,000 years ago (Turney et al., 2001) remain tentative, fire-event frequencies, estimated on the basis of macrocharcoal (>125 microns) and microcharcoal (10–125 microns) counts, increase in paleoenvironmental archives at Lake Euramoo and Quincan Crater from 4,000 years to peak at 2,000 years ago, following a period of dramatic rainforest expansion between 8,500 and 5,000 years ago (Haberle, 2005; Haberle et al., 2010). This coincides with increased Aboriginal site visibility and evidence for specialized use of rainforest resources in the region (Cosgrove et al., 2007). Accounts by Aboriginal Elders recount that frequent low-intensity burning enabled Aboriginal communities to maintain open “pockets” for camps and ceremonial activities and pathways between resource clusters and to ensure that sclerophyll and rainforest sub-canopies remained clear of unwanted rainforest taxa (Figure 2):

*“When we arrived at the camp, the women would clean up and burn the old leaves and sticks. They liked it nice and tidy in camp … Fires were built on top of old fires, each hut had at least one fireplace, and there was a large communal fire. Each family managed an area of rainforest, it was their responsibility to keep it clean and productive”* (M. Barlow, 2004, quoted in Ferrier, 2015).

Hill and Baird (2003) have also documented the use of fire by Rainforest Aboriginal People to convert patches of rainforest into open forest, to maintain patches of open forest and fire-enhanced resources within them, and to protect fire-sensitive resources in the rainforest and rainforest margins from wildfires. The extent to which burning of the rainforest was practiced in pre-contact times is also evident in the recent succession of sclerophyll environments, with an expansion of rainforest vegetation occurring following the forced suppression of fire activity over the last 100 years (Stanton et al., 2014a, 2014b). Although this process may also be influenced by climatic drivers (Cernusak et al., 2019), it is exacerbated by a lack of human management, which has resulted in the buildup of hazardous fuel loads and declines in numbers of economically useful plants and animals reliant on more open canopies (Van der Wal et al., 2009; Stanton et al., 2014a, 2014b).

**Figure 3:**
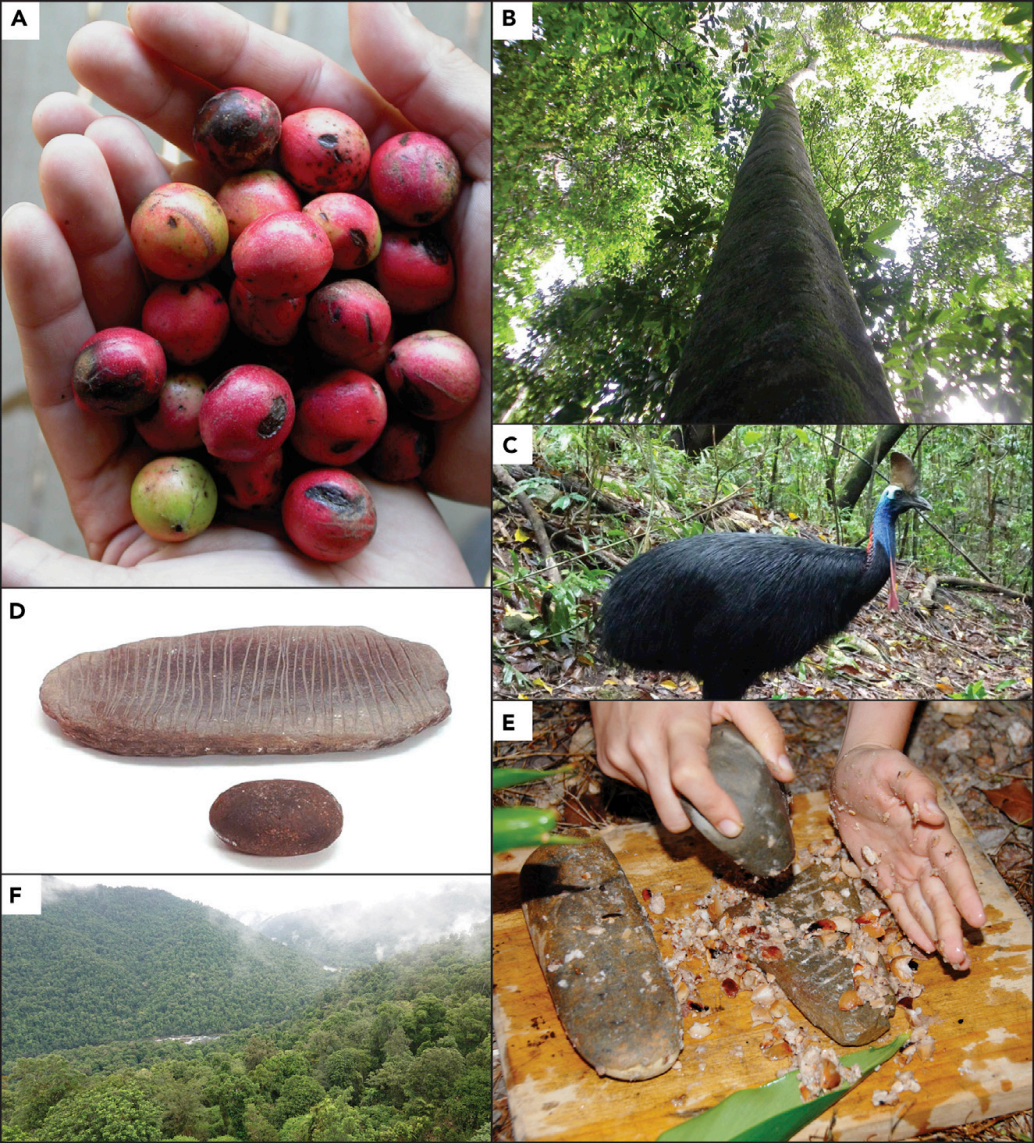
Compilation of rainforest foods and environments known to have been used by Rainforest Aboriginal groups (A) *Sundacarpus amara* (Black pine). Source: Photograph by R. Cosgrove. (B) *Beilschmiedia bancroftii* (Yellow walnut). Source: Photograph by R. Cosgrove. (C) *Casuarius casuarius* (cassowary). Source: Photograph by R. van Raders. (D) Incised slate grinding stone gifted to Mjöberg by a European settler in the Ravenshoe district. Source: Courtesy Museum of Ethnography, Stockholm. (E). Yellow walnut processing using traditional technology. Source: Photograph by R. Cosgrove. (F) Remnant tropical rainforest in the North Johnstone River district. Source: Photograph by R. Cosgrove.

Archeobotanical and archeological use-wear studies of stone tools have revealed the significance of various edible plants, many of which are part of the endemic Gondwanan heritage of the region and that would have been promoted by forest management, to Aboriginal diets and livelihoods across the Wet Tropics (Australian Department of Agriculture, Water and the Environment, 2021a). Archeobotanical evidence at sites such as Urumbal Pocket, Goddard Creek, Murubun rockshelter, and Jiyer Cave has shown the use of rainforest plants *c*. 5,000 years ago (Cosgrove, 1996; Cosgrove et al., 2007; King and Dotte-Sarout, 2018). Endocarps of yellow walnut (*Beilschmiedia bancroftii*) are particularly prominent after 2,000 years ago. Yellow walnuts are toxic and, as noted ethnographically, require leaching and processing, often using specialized basketry, to become edible (Pedley, 1992; Cosgrove, 1996; Tuechler et al., 2016). Starch residue analysis from incised grindstones suggests these tools were used to grind detoxified walnuts into paste (Cosgrove et al., 2007). A suite of other economically useful rainforest plants including black bean (*Castanospermum australe*), black walnut (*Endiandra palmerstonii*), black pine (*Sundacarpus amara*), cycads (*Cycas media*), and round yams (*Dioscorea bulbifera*) have also been recorded as part of the Aboriginal rainforest diet (Pedley, 1992; Cosgrove et al., 2007). Together, specialized use of starchy, high-protein plants would, in contrast to traditional assumptions of resource paucity, have enabled permanent occupation of the rainforest (Cosgrove, 1996; Wet Tropics Management Authority, 2020b; Australian Government Department of Agriculture, Water and the Environment, 2021a, 2021b) (Figure 3). This recognition has parallels with archeological and paleoecological research in the Amazon Basin where arguments that pre-colonial human populations could not thrive in the Amazon rainforest due to a lack of reliable carbohydrate access (Meggers, 1971) have now been proved to be inaccurate.

In the case of the Wet Tropics, it seems increasingly likely that these plants were also “cultivated,” at least in an agroforestry sense. Ethnographic and historical records note the caching of processing equipment (Mjöberg, 2015 [1918]) and storage of large, surplus piles of nuts in campsites (Tuechler et al., 2016). Ground-edge axes were likely a key part of the production of clearings for settlement, ceremonial grounds, and, perhaps, the promotion of economically beneficial tree species (Meston, 1904; Cosgrove, 1996; Cosgrove et al., 2007). This “planning” can also be seen in the Aboriginal management of animals. Zooarcheological analysis of sites such as Jiyer Cave have noted the exploitation of the green tail possum (*Pseudocherius archeri*), musky rat-kangaroo (*Hypsiprymnodon moschatus*), wallaby (*Macropod* family), and white-tailed rat (*Uromys caudimaculatus*), as well as snakes, birds, lizards, and fish (Cosgrove, 1996; Cosgrove and Raymont, 2002). Tree kangaroos (*Dendrolagus* spp.) were historically recorded as a highly sought-after flesh, hunted using tamed rainforest dingoes and elaborate rainforest lawyer cane (*Calamus* spp.) traps. Perhaps most intriguing are historical references to the Aboriginal capture and taming of young cassowaries (*Casuarius casuarius*) before the raising and fattening of these birds for later slaughter during large inter-tribal gatherings (O'Leary, 2019 [1918]) (Figure 3). As well as meat, given that these birds are known to concentrate fruit and nuts in their dung on the forest floor, an initially mutualistic relationship with this species may have been gradually transformed into more deliberate cultivation of economic plants, as noted in other sites of “agricultural origins” around the world (Spengler and Mueller, 2019).

## Historical ecology and ethnobotany

Crucial to modern ecological and conservation discussions is the *degree* to which these activities have left a mark on vegetation structure, distributions, and overall biodiversity. Research in the Amazon Basin over the last two decades has, for example, demonstrated the use of native and exogenous domesticated plants (Piperno and Pearsall, 1998; Clement et al., 2015; Lombardo et al., 2020) and vast landscape modifications (Maezumi et al., 2018a; Lombardo et al., 2020). However, perhaps most startling is the historical ecology and ethnobotanical research that has demonstrated that pre-colonial settlement has left a significant mark on the Amazon rainforest as it exists today (Levis et al., 2017; Maezumi et al., 2018a). For example, data from the Amazon Tree Diversity Network (ATDN) has shown that over 200 hyper-dominant species of the estimated 16,000 tree species of the Amazon account for half of the trees in the entire basin (Ter Steege et al., 2013). Species semi-domesticated or domesticated by Indigenous populations are five times more likely to be one of these 200 (Levis et al., 2017). Indeed, the location of these supposedly “wild” tree species, as well as their genetic makeup, have been definitively linked to human settlements, with humans evidently translocating key taxa (Clement et al., 2015; Shepard and Ramirez, 2011). Evidence points to dispersal across the basin of Brazil nut (*Bertholletia excelsa*) (Thomas et al., 2015), for example. In this way, pre-colonial populations have had a major hand in the current vegetation composition, distribution, fire regimes, and even carbon cycling across this vast basin (Fauset et al., 2015).

This research has forced ecologists and conservationists to contend with the Amazon Basin as a region of immense cultural, as well as natural, significance (Clement et al., 2015). Moreover, it has made the documentation of Indigenous knowledge of past land management essential to the formulation of sustainable policies for conservation (Cassino et al., 2019). Research on Aboriginal knowledge of past land use in the Wet Tropics can similarly provide valuable information on regionally specific traditions of tropical forest management, including fire management practices, toxic nut processing, and biocultural values. Nevertheless, evidence for past rainforest resource use has yet to be empirically connected to the distribution and composition of modern ecosystems. Botanical surveys have documented that economically important tree species are commonly found closely clustered together (Irvine, 1985). For example, within just a 0.5-hectare area of the Wet Tropics it was found that 20 trees of the Kuranda quandong (*Elaeocarpus bancroftii*) grew much closer together than would be expected as a product of natural seed dispersal (Tracey, 1982; Irvine, 1985). Indeed, concentrations of tree species used for their nuts and fruits across the Wet Tropics heritage area (Tracey, 1982; Irvine, 1985) have been ethnographically observed playing a key role in the structure of Aboriginal settlements, pathways, and even ceremonial practices (Harris, 1987). Today, the presence of economically useful trees are important indicators of archeological site locations and a cluster of economically useful tree species is usually discerned close to past locales of Aboriginal occupation (McCracken, 1989).

**Figure 4:**
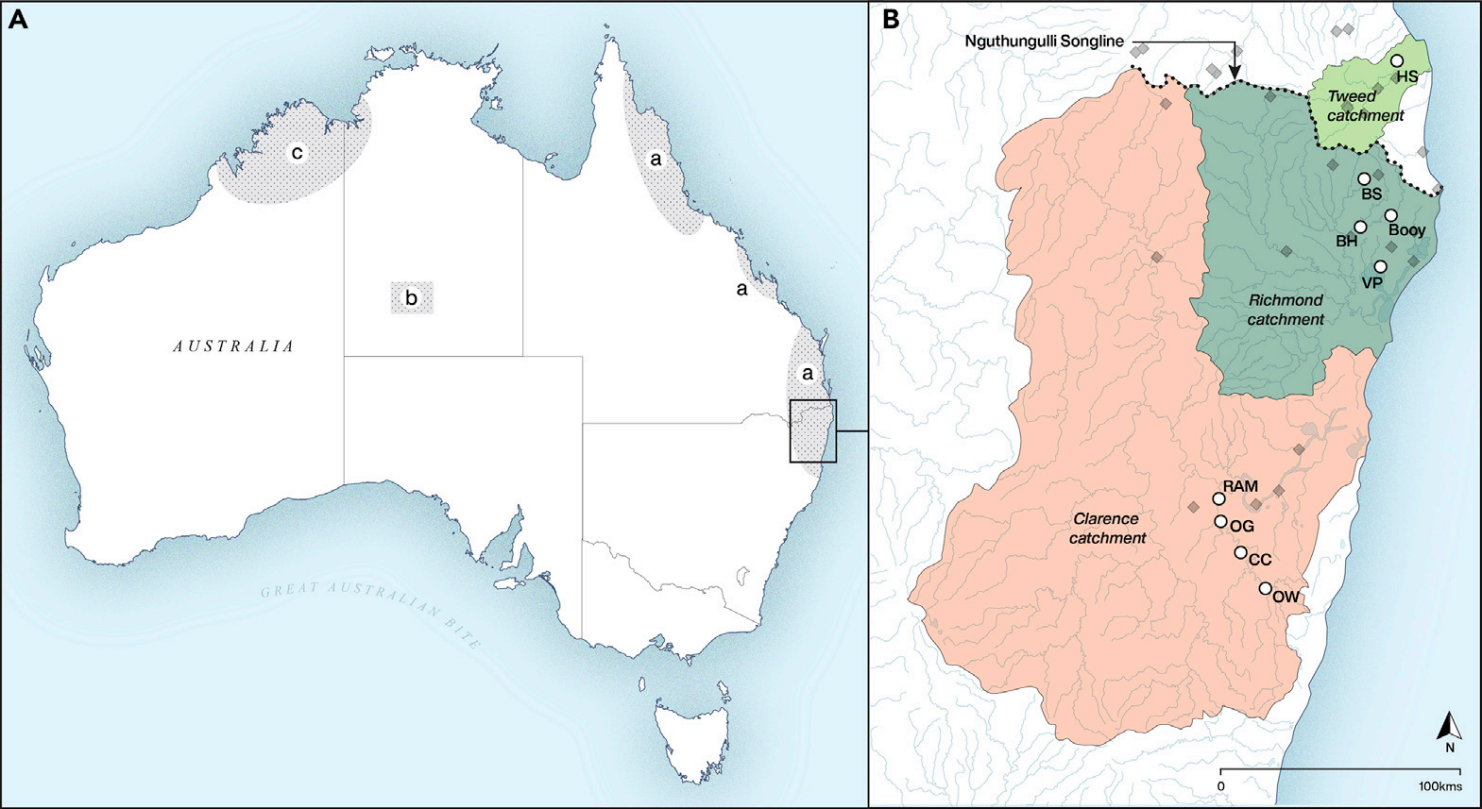
Evidence of pre-colonial anthropogenically influenced plant distributions in Australia (A) The areas of three recent genetic studies focusing on (a) black bean (*Castanospermum australe*) (Rossetto et al., 2017); (b) *Livistona mariae* (Kondo et al., 2012); and (c) boabab (*Adansonia gregorii*) (Rangan et al., 2015). (B) Detail of black bean known distribution based on herbarium records (diamonds), genetic samples (circles), and songline locations in northern New South Wales northern river catchments (adapted from Rossetto et al. (2017), Figure 2).

It thus seems likely that the distribution of food plants in the Wet Tropics are also, at least partly, a product of deliberate human agency. Aboriginal Elders and European settlers on the Atherton Tableland recall participating in and witnessing the traditional practice of spitting and spreading native seeds along walking tracks to ensure the continual renewal of food plants (Smith, 2007). Human-mediated plant distributions have been identified in several regions of Australia through genetic, ecological, linguistic, archeological, and ethnographic research (Figure 4). Baobab (*Adansonia gregorii*) and palms (*Livistona mariae*) have been shown to be anthropogenically dispersed species (Kondo et al., 2012; Rangan et al., 2015), the latter hypothesized to have appeared with humans in central Australia as early as 15,000 years ago. Research is also underway to test hypotheses of cultural imprints on key rainforest plants, and a similar multidisciplinary approach has now identified a recent and rapid dispersal of black bean (*Castanospermum australe*) based on its narrow genetic diversity and wide distribution across dissected territory in northern New South Wales (Figure 4) (Rossetto et al., 2017). Black bean is commonly found in economically useful tree clusters in the Wet Tropics, and Aboriginal oral histories and exchange networks provide a clear cultural framework for a distribution that is highly unlikely to be the result of natural dispersal mechanisms (Rossetto et al., 2017). This demonstrates the potential for innovative transdisciplinary research, with Aboriginal knowledge of landscape histories at its core, to further document the cultural heritage of the Wet Tropics Bioregion following trends in other tropical forest zones.

Trees also have clear cultural significance to a number of Aboriginal rainforest groups, notably seen in the form of dendroglyphs in eastern Australia, including the rainforest of the Wet Tropics (Buhrich et al., 2015; Buhrich and Murison, 2020), where they have been recorded on Mamu, Jirrbal, Western Yalanji, and Gimuy Yidinji estates. Rainforest dendroglyphs include abstract and geometric designs as well as a small number of male anthropomorphs and are often positioned on Aboriginal walking tracks (Buhrich et al., 2015; Buhrich and Murison, 2020). These carvings are often difficult to date and are increasingly under threat as trees die. In addition, Rainforest Aboriginal People have had restricted access to land and decision-making processes for a long time, limiting their ability to monitor them (Buhrich et al., 2015). Despite initially being considered impossible in the tropics due to a lack of environmental seasonality, dendrochronological studies have recently demonstrated that many tropical tree species do in fact form annual rings. Although many Australian species have yet to be tested for dendrochronological potential, species such as Australian red cedar (*Toona ciliata*) have been successfully used to reconstruct precipitation and forest dynamics (Heinrich et al., 2008). Such analyses can be used to develop chronologies for tree growth and dendroglyph creation, as well as to study changes in growth patterns of different species with different ecological tolerances and, ultimately, changing human presence and use of the forest (Caetano-Andrade et al., 2019).

## The necessity of an active cultural heritage—a celebration of “On Country” management in the Wet Tropics

**Figure 5:**
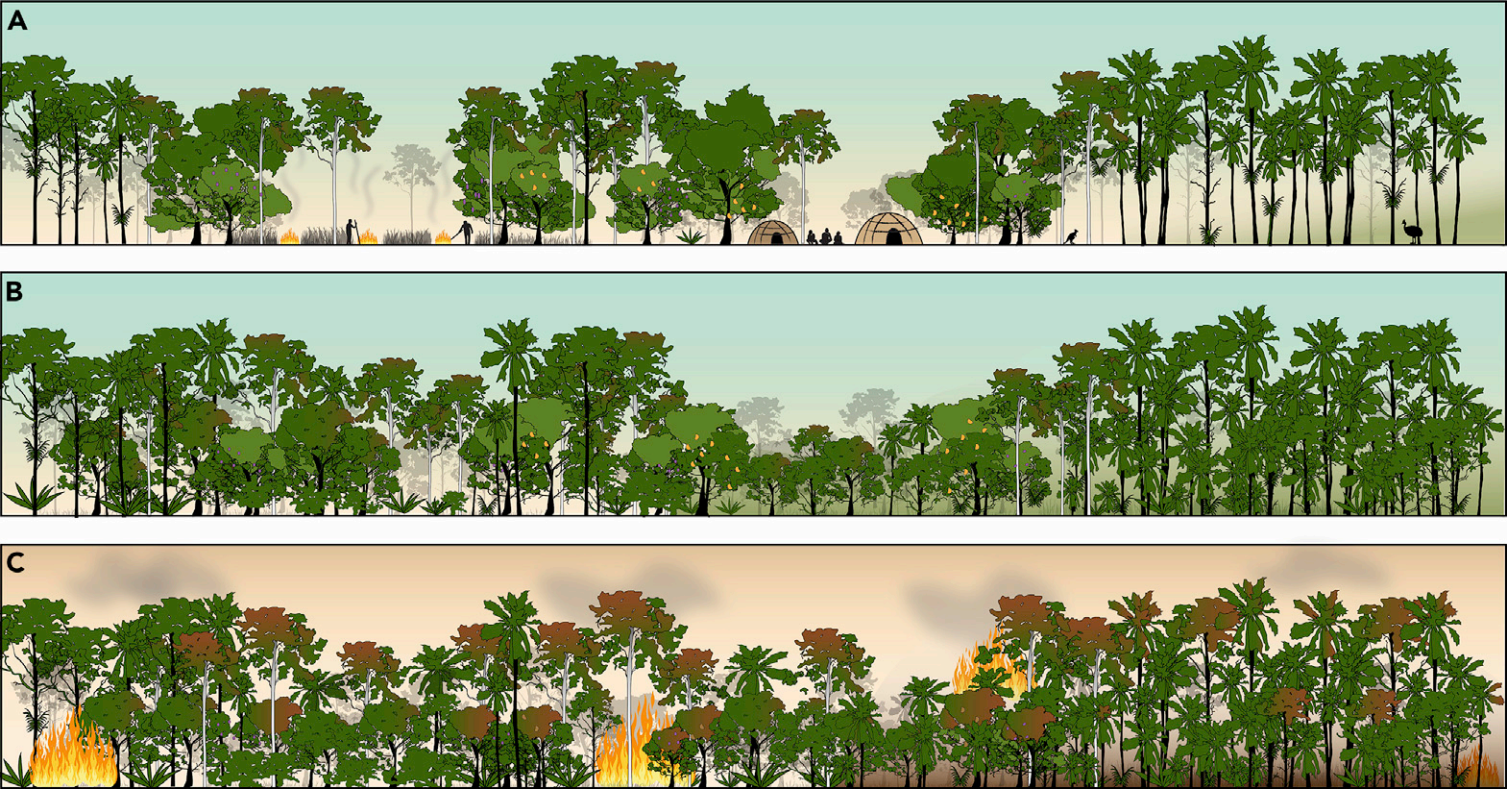
Schematic showing changing fire dynamics following the disruption of Aboriginal burning practices within the rainforests of the Wet Tropics (A) Pre-1900: Pre-European Indigenous land management in the Wet Tropics Bioregion using fire to maintain open pockets for camp-sites, clear pathways, rainforest/sclerophyll edges, and intact, biodiverse rainforest areas with managed floors (B). Early to mid-1900s: Thickening of rainforest under-growth and invasion of rainforest species into sclerophyll pockets and rainforest/sclerophyll frontiers after restriction of Rainforest Aboriginal People from full-time occupation of country (C). Post 1940s: Disruptions to fire and Aboriginal land management resulting in dense under-growth, which, with human-induced climate warming and aridity, is leading to growing numbers of unprecedented wildfires.

The emerging archeological, historical, and paleoecological data further highlights the necessity of including Traditional Owners in management plans for the environments of the Wet Tropics, something that they, and their supporters, have been campaigning for since the inception of the Wet Tropics Bioregion, and that is being increasingly achieved through the National Heritage listing for cultural values and Aboriginal representation within the Wet Tropics Management Authority (Review Steering Committee, 1998; Wet Tropics Aboriginal Cultural and Natural Plan Project Team, 2005; Wet Tropics Management Authority 2020e). The cessation of Indigenous burning in the Amazon Basin changed forest fire regimes, from low-intensity, frequent burns to large-scale European clearance or abandonment, leading to a loss of fire-adapted taxa and the build-up of flammable fuel load on the forest floor (Maezumi et al., 2018b). With previous substantial barriers to Indigenous management for nearly 50 years, similar dynamics have been observed in the rainforests of the Wet Tropics (Stanton et al., 2014a, 2014b). As Figure 5 shows, long-term Aboriginal landscape structuring in the form of cleared pathways, open “pockets” for settlement, rainforest/sclerophyll edges, useful clusters of economic trees species, and intact, biodiverse rainforest areas with managed floors would once have acted as key barriers to the spread of fire (Figure 5A). However, these managed landscapes have been compromised as rainforest species encroach sclerophyll ecosystems in the absence of well-honed human management practices (Figure 5B). Moreover, sub-canopy growth that was once kept clear by frequent Aboriginal burns represents an increased fuel load that, when accompanied by ever drier conditions in the face of human-induced climate change, can ignite and destroy even the most fire-adapted of tree species (Figure 5C). Increasingly, severe forest fires are now affecting rainforest (Gergis, 2019; Edwards and Krockenberger, 2006), and food trees once promoted by Aboriginal communities will soon be succeeded by other rainforest tree taxa (Stanton et al., 2014a, 2014b; Van der Wal et al., 2009). It is not just the native flora that is at risk, however. Tree kangaroos thrive in forests with an open understory (Heise-Pavlov et al., 2011; Heise-Pavlov, 2017); meanwhile cassowaries require some form of open “corridors” and patches for feeding (Kutt et al., 2004; Campbell et al., 2014). Consequently, frequent Aboriginal clearance of understories and tracks, alongside maintenance and protection of areas of rainforest, would have benefited populations of these endemic rainforest animals (Hill and Baird, 2003). The regrowth of dense vegetation in the absence of Aboriginal land management, alongside urbanization, expanding agriculture, vast areas of complete forest clearance, and the incursion of invasive species, such as feral cats (Laurance and Goosem, 2008; Stork et al., 2008), are posing a serious conservation risk to these, and other, endemic rainforest animals.

These issues have been repeatedly emphasized by Rainforest Aboriginal Peoples, as well as their administrative and scientific supporters, and are now being increasingly acted upon. The Wet Tropics Management Authority now promotes rainforest Aboriginal culture through the provision of a small grants program for Rainforest Aboriginal People to conduct research and cultural heritage protection, the circulation of a Rainforest Aboriginal Newsletter (Wet Tropics Management Authority, 2020c), dedicated Aboriginal project staff, support for Indigenous Land and Sea Rangers, and a commitment to Aboriginal engagement in the review of the Wet Tropics Management Plan overseen by the Traditional Owner Leadership Group (Cultural Values Project Steering Committee, 2016; Wet Tropics Management Authority, 2020d, 2020e). Under the recently announced “Wet Tropics Strategic Plan 2020-2030,” Rainforest Aboriginal People can, among other things, seek joint management of National Parks and have input into the permit process for scientific research on their native title estates. The Strategic Plan also addresses access arrangements that aim to support Rainforest Aboriginal People contributing to ongoing research. These approaches are already showing clear benefits, both ecological and cultural. In the Daintree National Park, north of the city of Cairns, Kuku Yalanji Bama Traditional Owners, with the support of Queensland Parks and Wildlife Services, are now applying cultural burns to protect life and property, mitigate wildfires, and maintain the natural diversity of the region, and Aboriginal traditional knowledge is being applied to develop fire management zones to protect cultural landscapes as a matter of high priority (Queensland Parks and Wildlife Service, Department of Environment and Science, 2019). Since 2017, the Queensland Department of Environment and Science has funded a number of Aboriginal-led fire projects in the Wet Tropics through their Looking After Country grant program. One is that of the Djabugay Bulmba Rangers, who have recently developed an active annual program of managing country with fire. This involves renewed studying of landscapes, understanding which forest types occur where, and when it is the right time to apply fire as indicated by seasonal signs in the vegetation (Hunter, 2020). The aim is not simply to mechanically reduce fuel loads that have been causing significant problems during wildfire events, but to culturally reconnect with the landscape (Hunter, 2020). The reapplication of cultural burning has seen the Djabugay find burning-related meanings in songs and phrases that were previously lost, while also rejuvenating the use of their language while working on country. To the Djabugay Bulmba Rangers, cultural burning also provides a source of economic benefits through the carbon market for the group, while they simultaneously make positive changes to the environment. Federal initiatives relating to the establishment of Indigenous Protected areas also hold much potential for the Wet Tropics (National Environmental Research Program, 2014).

There has also been growing support for Aboriginal Ranger Groups, such as that of the Djabugay, as well as the Jabalbina and Girrigun, from the Queensland State, Australian Federal Governments, and the Wet Tropics Management Authority. These groups enable Rainforest Aboriginal People to take an active role in monitoring ecological dynamics “*On Country*” and managing vegetation zones and animal species. These initiatives are positive steps forward and are a testament to the tireless campaigning by Rainforest Aboriginal People. Significantly, the Wet Tropics Strategic Plan 2020–2030 now allows for a number of landscape uses within the World Heritage Area, including permanent living areas. The Mandingalbay Yidinji People have developed a certified ecotourism initiative as part of their native title while also monitoring their Indigenous Protected Area, linked with the International Union of the Conservation of Nature, through Ranger Groups and biodiversity surveys (Mandingalbay Yidinji People, 2017). These successful initiatives clearly indicate that more funding is required for these benefits and practices to be extended more widely across the Wet Tropics. Part of this requires the enabling of Traditional Owners to look after country, which they currently describe as “sick” or “dirty,” through burning practices mentioned above, and the re-opening of old walking tracks and sclerophyll forest pockets. This process requires understanding of individual groups' “estates” within the Wet Tropics, individual Traditional Owner groups' aspirations for land management, and how those aspirations match the myriad of policy and legislative requirements. Although regional leadership groups and formal alliances play a role in negotiations with government bodies, communities' desires to make decisions at the Prescribed Body Corporate (PBC) level, which reflects native title engagement and Indigenous governance in land management, matters. It is also important to recognize that cultural heritage is broader than purely material remains and that for Rainforest Aboriginal Peoples, complex knowledge systems, which are often associated with active relationships between people and places, are equally important (Bottoms, 1999; Pannell, 2005; Gratani et al., 2014).

Based on the information reviewed above, we would suggest that “long-term” perspectives from archeology, history, and paleoecology can also make contributions to these management plans by adding detailed reconstructions of vegetation change, insights into human use of plants and animals, and the mapping of cultural sites and walking routes, into the existing corpus of Traditional Owner knowledge. This is something the Wet Tropics Management Authority is also increasingly supporting. In September 2019, the Queensland Government “Looking After Country” grant supported Jirrbal Traditional Owners to return to Urumbal Pocket on the upper Tully River (Figure 2). As part of the visit, Jirrbal Traditional Owners undertook archeological, botanical, faunal, and cultural surveys, working with archeologists who have previously excavated the site to demonstrate occupation back to ~8,000 years ago (Cosgrove et al., 2007; Ferrier, 2015) and the subsequent development of toxic nut food processing at the site. Meanwhile, ecologists and historical ecologists identified the imprint left on the local vegetation by past human activities and subsequent management limitation. The visit facilitated a reconnection between Traditional Owners and their country and initiated discussions relating to the application of burning and other land management tools to re-open the pocket for settlement and activities as well as pathways between this pocket and other areas of the landscape (Wet Tropics Management Authority, 2019). Further support of these varied examples of Indigenous-led projects will help yield country-specific approaches to land management, custodianship, ecological dynamics and sustainability, and the reinvigoration of knowledge systems connected to active management of landscapes that were significantly impacted as a product of colonial invasion and Aboriginal relocation, as has been fore-fronted in other parts of Australia (Fletcher et al., 2020).

## Conclusions: The global cultural and natural significance of the Wet Tropics

In the last two decades, there has been a dramatic and prominent re-evaluation of past human influences on tropical rainforests around the world (Iriarte et al., 2007; Clement et al., 2015; Levis et al., 2017; Roberts et al., 2017a; Roberts, 2019; Maezumi et al., 2018a). In the 1980s, anthropologists argued that tropical rainforest environments were effective “Green Deserts,” being carbohydrate poor and lacking reliable protein resources (Bailey et al., 1989; Bailey and Headland, 1991). Archeologists drew on these ideas, as well as challenges related to disease and thermoregulation in these habitats, to suggest that they would have also represented significant barriers to human occupation, at least until the development of specialized subsistence technologies during the Holocene (Gamble, 1993; Bird et al., 2005; Boivin et al., 2013). Nonetheless, these perceptions have been refuted by anthropologists and historical ecologists (e.g., Colinvaux et al., 1991), as well as through growing archeological and paleoecological research that has highlighted that our species, *Homo sapiens*, rapidly occupied tropical rainforest environments as it arrived in South Asia, Southeast Asia, and Near Oceania (Barker et al., 2007; Summerhayes et al., 2010; Roberts et al., 2015, 2017b; Westaway et al., 2017). Not only that, but from the Holocene, these environments witnessed the emergence of cultivation practices (Denham et al., 2003; Lombardo et al., 2020), vast urban centers (Evans et al., 2007; Canuto et al., 2018), and the often-catastrophic impacts of European colonialism (Lewis and Maslin, 2015; Koch et al., 2019). Today, it has been recognized that instead of being “blanks” on an archeological map, pre-industrial human populations living in tropical forests were so significant that they dictated the nature of the soils (Neves et al., 2003; Arroyo-Kalin, 2010, 2012), species diversity and distribution (Ter Steege et al., 2013; Clement et al., 2015; Levis et al., 2017), and perhaps even regional (Cook et al., 2012) and global (Koch et al., 2019) climates, making them key sites of discussions of the origins of the “Anthropocene” whose legacies we see all around us today (Roberts et al., 2018).

The Wet Tropics Bioregion has long been acknowledged as being of global significance to ecological and evolutionary studies (Sanderson, 2008; Wet Tropics Management Authority, 2020a). However, in light of the ongoing work, activism, and expression of traditional knowledge by Rainforest Aboriginal Peoples, and our review of the existing multidisciplinary evidence, the Wet Tropics Bioregion is also of clear global significance for its cultural values (see also Hill et al., 2011). Although the tempo of occupation may be different to other tropical forests (Roberts and Petraglia, 2015), and archeological evidence for Pleistocene occupation remains sparse, Traditional Owner knowledge suggests habitation by at least the terminal Pleistocene (Dixon, 1983; Pannell, 2008), with definite material traces of occupation by 8,000 years ago (Cosgrove et al., 2007; Ferrier, 2015). Detailed archeobotanical, archeozoological, archeological, and paleoecological evidence, as well as persisting knowledge and testimonies of Rainforest Aboriginal Peoples, documents the expansion of populations, sophisticated processing of toxic rainforest tree fruits, the burning of rainforest and sclerophyll environments, and hunting of forest-dwelling vertebrates during the last 2,000 years (Hill and Baird, 2003; Cosgrove et al., 2007; Ferrier, 2015). National bodies and heritage statements are now rightly using this evidence to recognize the clear cultural presence of Rainforest Aboriginal Peoples in the Wet Tropics, and the importance of their custodianship over these landscapes today. To this, we would also add, based on the material covered above, that there are hints of evidence for active translocation of certain tree species, management of economic useful plant distributions, and at least some tentative signals of animal management. We would also encourage a reconsideration of the scale of pre-colonial Aboriginal presence in the region, with existing estimates based on European census data likely to be skewed by the same biases that have plagued population estimates across Australia (Gammage, 2012; Pascoe, 2018). Based on ethnohistorical accounts and archeological artifact distributions, it seems likely that the number of Aboriginal people living within the Wet Tropics, making use of its plant and animal resources, and shaping these ecosystems, was much more significant than has often been appreciated. A re-evaluation of the demographic impacts of colonial invasion, relocation, and genocide in the Wet Tropics is likely to yield significant insights into the historical ecology of the region, such as those found elsewhere in the tropics, for example, in the Amazon Basin (e.g., Heckenberger et al., 2003, 2008), much of the Neotropics (Koch et al., 2019), and the Pacific realm (Newson, 2009).

UNESCO has recently highlighted the significance of joint listings in tropical forest landscapes. We hope that the long-term, multidisciplinary perspectives presented in this review, based on the existing literature, adds further resources for the ongoing knowledge contributions and campaigning by Rainforest Aboriginal Peoples and their supporters, to re-list the Wet Tropics Bioregion as a site of international biocultural (cultural and natural) heritage with UNESCO (Cultural Values Project Steering Committee, 2016). This would add to the two Indigenous cultural landscapes formally recognized as joint sites of cultural and natural heritage by UNESCO in Australia: Budj Bim in Victoria and Uluru-Kata Tjuta in Central Australia. The Wet Tropics has now been acknowledged nationally for both its cultural and natural values (Review Steering Committee, 1998; Wet Tropics Aboriginal Cultural and Natural Plan Project Team, 2005; Cultural Values Project Steering Committee, 2016), and, moreover, Rainforest Aboriginal Peoples are increasingly applying traditional knowledge and land management practices, including burning, with ever-growing support from administrative bodies such as the Queensland Department of Environment and Science. The provision for more specific “Country Based Plans” for all Rainforest Aboriginal groups, and the development and support of more detailed local paleoecological, archeological, and anthropological assessments of land management changes, within a framework of traditional knowledge, will almost certainly, like other locally driven conservation schemes around the tropics, yield profitable results (Ricketts et al., 2010; Sheil et al., 2015). Australia is facing intensifying challenges in the form of climate change, more frequent bushfires, and a loss of biodiversity that are only likely to increase as the 21^st^ century wears on. In the Wet Tropics, these challenges are not only beginning to impact the unique flora and fauna that are so crucial to its international natural heritage listing, but also the millennia of human occupation, cultural values, and knowledge caught up in its biota and land. Growing acknowledgment, and detailed investigation, of the joint natural-cultural significance of this region, so long advocated by its Traditional Owners, promises to further raise awareness not only about the international importance of the Wet Tropics but also as to how to best shape and inform the protection of the ecological, economic, and cultural resources that are so crucial for its Aboriginal inhabitants as well as for the nation, and the planet, as a whole.

## References

[bib1] Anderson C., Robins R., Mehan B., Jones R. (1988). ‘Dismissed due to lack of evidence? Contemporary Kuku- Yalangi campsites and the archaeological record’. Archaeology with Ethnography: An Australian Perspective.

[bib2] Arroyo-Kalin M. (2010). The Amazonian Formative: crop domestication and anthropogenic soils. Diversity.

[bib3] Arroyo-Kalin M. (2012). Slash-burn-and-churn: landscape history and crop cultivation in pre-Columbian Amazonia. Quat. Int..

[bib4] Australian Government Department of Agriculture, Water and the Environment (2021). Wet Tropics of Queensland. http://www.environment.gov.au/cgi-bin/ahdb/search.pl?mode=place_detail;place_id=105689.

[bib5] Australian Government Department of Agriculture, Water and the Environment (2021). World Heritage Places – Wet Tropics of Queensland. https://www.environment.gov.au/heritage/places/world/wet-tropics.

[bib6] Bailey R., Head G., Jenike M., Owen B., Rechtman R., Zechenter E. (1989). Hunting and gathering in tropical rain forest; Is it possible?. Am. Anthropol..

[bib7] Bailey R.C., Headland T.N. (1991). The tropical rainforest: is it a productive environment for human foragers?. Hum. Ecol..

[bib8] Barber M., Jackson S. (2015). Remembering ‘the blackfellows’ dam’: Australian Aboriginal water management and settler colonial riparian law in the upper Roper River, Northern Territory. Settl. Colon. Stud..

[bib9] Barker G., Barton H., Bird M., Daly P., Datan I., Dykes A., Farr L., Gilbertson D., Harrisson B., Hunt C. (2007). The ‘human evolution’ in lowland tropical Southeast Asia: the antiquity and behaviour of anatomically modern humans at Niah Cave (Sarawak, Borneo). J. Hum. Evol..

[bib10] Beale E. (1977). Kennedy of Cape York.

[bib11] Bird M., Taylor D., Hunt C. (2005). Palaeoenvironments of insular Southeast Asia during the last glacial period: a savanna corridor in Sundaland?. Quat. Sci. Rev..

[bib12] Boivin N., Fuller D.Q., Dennell R., Allaby R., Petraglia M.D. (2013). Human dispersal across diverse environments of Asia during the upper Pleistocene. Quat. Int..

[bib13] Bottoms T. (1999). Djabugay Country: An Aboriginal History of Tropical North Queensland.

[bib14] Bowman D.M.J.S., Cook G.D. (2001). Can stable carbon isotopes (delta13C) in soil carbon be used to describe the dynamics of Eucalyptus savanna-rainforest boundaries in the Australian monsoon tropics?. Austral. Ecol..

[bib15] Buhrich A., Ferrier Å., Grimwade G. (2015). Attributes, preservation and management of dendroglyphs from the Wet Tropics rainforest of Northeast Australia. Aust. Archaeol..

[bib17] Buhrich A., Murison J. (2020). The Western Yalanji dendroglyph: the life and death of an Aboriginal carved tree. J. Commun. Archaeol. Heritage.

[bib18] Builth H., Kershaw A.P., White C., Roach A., Hartney L., McKenzie M., Lewis T., Jacobsen G. (2008). Environmental and cultural changes on the Mt Eccles lava-flow landscapes of southwest Victoria, Australia. Holocene.

[bib19] Bush M.B., McMichael C.H., Piperno D.R., Silman M.R., Barlow J., Peres C.A., Power M., Palace M.W. (2015). Anthropogenic influence on Amazonian forests in pre-history: an ecological perspective. J. Biogeogr..

[bib20] Caetano-Andrade V.L., Clement C.R., Weigel D., Boivin N., Schöngart J., Roberts P. (2020). Tropical trees as time capsules of anthropogenic activity. Trends Plant Sci..

[bib21] Campbell H.A., Dwyer R.G., Fitzgibbons S., Klein C.J., Lauridsen G., McKeown A., Olsson A., Sullivan S., Watts M.E., Westcott D.A. (2014). Prioritising the protection of habitat utilised by southern cassowaries *Casuarius casuarisu johnsonii*. Endangered Speices Res..

[bib22] Campbell J.B. (1984). Extending the archaeological frontier: a review of work on the prehistory of north Queensland. Qld. Archaeol.Res..

[bib23] Canuto M.A., Estrada-Belli F., Garrison T.G., Houston S.D., Acuña M.J., Kovác M., Marken D., Nondédéo P., Auld-Thomas L., Castanet C. (2018). Ancient lowland Maya complexity as revealed by airborne laser scanning of northern Guatemala. Science.

[bib24] Cassino M.F., Alves R.P., Levis C., Watling J., Junqueira A.B., Shock M.P., Ferreira M.J., Caetano Andrade V.L., Furquim L.P., Coelho S.D., Alberquerque U., de Lucena R., Cruz da Cunha L., Alves R. (2019). Ethnobotany and ethnoecology applied to historical ecology. Methods and Techniques in Ethnobiology and Ethnoecology.

[bib25] Cernusak L.A., Haverd V., Brendel O., Le Thiec D., Guehl J.-M., Cuntz M. (2019). Robust response of terrestrial plants to rising CO_2_. Trends. Plant Sci..

[bib26] Clement C.R., Denevan W.M., Heckenberger M.J., Junqueira A.B., Neves E.G., Teixera W.G., Woods W.I. (2015). The domestication of Amazonia before European conquest. Proc. R. Soc. B Biol. Sci..

[bib27] Colinvaux P.A., Bush M.B. (1991). The rain-forest ecosystem as a resource for hunting and gathering. Am. Anthropol..

[bib28] Cook B.I., Anchukaitis K.J., Kaplan J.O., Puma M.J., Kelley M., Gueyffier D. (2012). Pre-Columbian deforestation as an amplifier of drought in Mesoamerica. Geophys. Res. Lett..

[bib29] Cosgrove R. (1996). Past human use of rainforests: an Australasian perspective. Antiquity.

[bib30] Cosgrove R. (2005). Coping with noxious nuts. Nat. Aust..

[bib31] Cosgrove R., Raymont E. (2002). Jiyer Cave revisited: preliminary results from northeast Queensland rainforest. Aust. Archaeol..

[bib32] Cosgrove R., Field J., Ferrier Å. (2007). The archaeology of Australia’s tropical rainforests. Palaeogeogr. Palaeoclim. Palaeoecol..

[bib33] Cultural Values Project Steering Committee (2016). Which Way Australia’s Rainforest Culture: Relisting the Cultural Values for World Heritage. Discussion Paper about Realising the National and International Recognition of the Rainforest Aboriginal Cultural Values of the Wet Tropics Region and World Heritage Area..

[bib150] David B., Chant D. (1995). Rock Art and Regionalisation in North Queensland Prehistory. Memoirs of the Queensland Museum 37: Part.

[bib36] Denham T.P., Haberle S.G., Lentfer C., Fullagar R., Field J., Therin M., Porch N., Winsborough B. (2003). Origins of agriculture at kuk swamp in the highlands of new Guinea. Science.

[bib37] Dixon R.M.W. (1983). Searching for Aboriginal Languages. Memoirs of a Field Worker.

[bib38] Edwards W., Krockenberger A. (2006). Seedling mortality due to drought and fire associated with the 2002 El Nino Event in a tropical rainforest in north-east Queensland, Australia. Biotropica.

[bib39] Evans D., Pottier C., Fletcher R., Hensley S., Tapley I., Milne A., Barbetti M. (2007). A comprehensive archaeological map of the world’s largest preindustrial settlement complex at Angkor, Cambodia. Proc. Natl. Acad. Sci. U S A.

[bib40] Fauset S., Johnson M.O., Gloor M., Baker T.R., Monteagudo M.A., Brienen R.J.W., Feldpausch T.R., Lopez-Gonzalez G., Malhi Y., ter Steege H. (2015). Hyperdominance in Amazonian forest carbon cycling. Nat. Communs..

[bib41] Ferrier Å. (2015).

[bib42] Fletcher M.-S., Hall T., Alexandra A.N. (2020). The loss of an indigenous constructed landscape following British invasion of Australia: an insight into the deep human imprint on the Australian landscape. Ambio.

[bib43] Gamble C. (1993). Timewalkers: The Prehistory of Global Colonization.

[bib44] Gammage B. (2012). The Biggest Estate on Earth.

[bib45] Garnett S.T., Burgess N.D., Fa J.E., Fernández-Llamazares Á., Molnár Z., Robinson C.J., Watson J.E.M., Zander K.K., Austin B., Brondizio E.S. (2017). A spatial overview of the global importance of Indigenous lands for conservation. Nat. Sustain..

[bib46] Gergis J. (2019). I Never Thought I’d See the Australian Rainforest Burning. What Will it Take for Us to Wake up to the Climate Crisis. https://www.theguardian.com/commentisfree/2019/sep/10/i-never-thought-id-see-the-australian-rainforest-burning-what-will-it-take-for-us-to-wake-up-to-the-climate-crisis.

[bib47] Graham A.W. (2006). The CSIRO Rainforest Permanent Plots of North Queensland.

[bib48] Gratani M., Bohensky E.L., Butler J.R.A., Sutton S.G., Foale S. (2014). Experts’ perspectives on the integration of Indigenous knowledge and science in Wet Tropics natural resource management. Aust. Geographer..

[bib49] Haberle S. (2005). A 23,000-year pollen record from Lake Euramoo, wet tropics of NE Queensland, Australia. Quat. Res..

[bib50] Haberle S.G., Rule S., Roberts P., Heijnis H., Jacobsen G., Turney C., Cosgrove R., Ferrier A., Moss P., Mooney S., Kershaw P. (2010). Paleofire in the wet tropics of northeast Queensland, Australia. PAGES.

[bib51] Hallam S.J. (1989). Fire and Hearth: A Study of Aboriginal Usage and European Usurpation in South-Western Australia.

[bib52] Harrington G.N., Sanderson K.D. (1994). Recent contraction of wet sclerophyll forest in the wet tropics of Queensland due to invasion by rainforest. Pac. Conserv. Biol..

[bib53] Harris D., Blurton-Jones N.G., Reynolds V. (1978). Adaptation to a tropical rain-forest environment: Aboriginal subsistence in northeast Queensland. Human Behaviour and Adaptation.

[bib54] Harris D., Harris M., Ross E.B. (1987). Aboriginal subsistence in a tropical rain forest environment: food procurement, cannibalism, and population regulation in northeastern Australia. Food and Evolution: Toward a Theory of Human Food Habits.

[bib55] Heckenberger M.J., Kuikuro A., Kuikuro U.T., Russell J.C., Schmidt M., Fausto C., Franchetto B. (2003). Amazonia 1492: pristine forest or cultural parkland?. Science.

[bib56] Heckenberger M.J., Russell J.C., Fausto C., Toney J.R., Schmidt M.J., Pereira E., Franchetto B., Kuikuro A. (2008). Pre-Columbian urbanism, anthropogenic landscapes, and the future of the Amazon. Science.

[bib57] Heinrich I., Weidner K., Helle G., Vos H., Banks J.C.G. (2008). Hydroclimatic variation in Far North Queensland since 1860 inferred from tree rings. Palaeogeogr. Palaeoclimatol. Palaeoecol..

[bib58] Heise-Pavlov S. (2017). Current knowledge of the behavioural ecology of Lumholtz’s tree-kangaro (Dendrolagus lumholtzi). Pac. Conserv. Biol..

[bib59] Heise-Pavlov S., Jackrel S.L., Meeks S. (2011). Conservation of a rare arboreal mammal: habitat preferences of the Lumholtz’s tree-kangaroo, *Dendrolagus lumholtzi*. Aust. Mammal..

[bib60] Hilbert D.W., Graham A., Hopkins M.S. (2007). Glacial and interglacial refugia within a long-term rainforest refugium: the Wet Tropics Bioregion, NE Queensland, Australia. Palaeogeogr. Palaeoclimatol. Palaeoecol..

[bib61] Hill R., Griggs P., Bamanga Bubu Ngadimunku Incorporated (2000). Rainforest, agriculture and aboriginal fire-regimes. Aust. Geogr. Stud..

[bib62] Hill R., Baird A. (2003). Kuku-Yalanji rainforest Aboriginal people and carbohydrate resource management in the wet tropics of Queensland, Australia. Hum. Ecol..

[bib63] Hill R., Baird A., Buchanan D., Denman C., Fischer P., Gibson K., Johnson J., Kerry A., Kulka G., Madsen E. (2004). Yalanji-Warranga Kaban: Yalanji People of the Rainforest Fire Management Book.

[bib64] Hill R., Cullen-Unsworth L.C., Talbot L.D., McIntyre-Tamwoy S. (2011). Empowering Indigenous peoples’ biocultural diversity through World Heritage cultural landscapes: a case study from the Australian humid tropical forests. Int. J. Heritage Stud..

[bib65] Horsfall N., Veth P., Hiscock P. (1996). Holocene occupation of the tropical rainforests of North Queensland. Archaeology of Northern Australia.

[bib66] Hunter B. (2020). Talking on Country – Looking after Country, Aboriginal Land Management. http://barryjhunter.blogspot.com/2020/01/where-does-indigenous-land-management.html.

[bib67] Iriarte J., Denham T., Vrydaghs L. (2007). Rethinking Agriculture: Archaeological and Ethnoarchaeological Perspectives.

[bib68] Irvine A.K., Jones G.P. (1985). *Commercial prospects for edible nuts of* athertonia diversifolia *(C.T. White), L. Johnson & briggs (proteaceae) and Elaeocarpus bancroftii F. Muell & F.M. Bailey (Elaeocarpaceae*).

[bib69] Jones R. (1979). The fifth continent: problems concerning the human colonization of Australia. Annu. Rev. Anthropol..

[bib70] Jurskis V. (2009). River red gum and white cypress forests in south-western New South Wales, Australia: ecological history and implications for conservation of grassy woodlands. For. Ecol. Management.

[bib71] Kemp J.E., Lovatt R.J., Bahr C., Kahler C.P., Appelman C.N. (2007). Pre-clearing vegetation of the coastal lowlands of the wet tropics bioregion, North Queensland. Cunninghamia.

[bib72] Kershaw A.P. (1986). The last two glacial-interglacial cycles from northeastern Australia: implication for climate change and Aboriginal burning. Nature.

[bib73] Kershaw A.P., Bretherton S.C.L., van der Kaars W.A.( (2007). A complete pollen record of the last 230 ka from Lynch's Crater, north-eastern Australia. Palaeogeogr. Palaeoclimatol. Palaeoecol..

[bib74] King F., Dotte-Sarout E. (2018). Wood charcoal analysis in the in tropical rainforest: a pilot study identifying firewood used at toxic nut processing sites in Northeast Queensland, Australia. Veg. Hist. Archaeobot..

[bib75] Koch A., Brierley C., Maslin M.M., Lewis S.L. (2019). Earth systems impacts of the eruopean arrival and great dying in the americas after 1492. Quat. Sci. Rev..

[bib76] Kondo T., Crisp M.D., Linde C., Bowman D.M.J.S., Kawamura K., Kaneko S., Isagi Y. (2012). Not an ancient relic: the endemic *Livistonia* palms of arid central Australia could have been introduced by humans. Proc. Biol. Soc..

[bib77] Kutt A.S., King S., Garnett S.T., Latch P. (2004). Distribution of Cassowary Habitat in the Wet Tropics Bioregion, Queensland.

[bib78] Laurance W.F., Goosem M., Stork N.E., Turton S.M. (2008). Impacts of habitat fragmentation and lineal clearings on Australian rainforest biota. Living in a Dynamic Tropical Forest Landscape.

[bib79] Levis C., Costa F.R.C., Bongers F., Peña-Claros M., Clement C.R., Junqueira A.B., Neves E.G., Tamanaha E.K., Figueiredo F.O.G., Salomão R.P. (2017). Persistent effects of pre-Columbian plant domestication on Amazonian forest composition. Science.

[bib80] Lewis S.L., Maslin M.A. (2015). Defining the anthropocene. Nature.

[bib81] Lombardo U., Iriarte J., Hilbert L., Ruiz-Pérez J., Capriles J.M., Veit H. (2020). Early Holocene crop cultivation and landscape modification in Amazonia. Nature.

[bib82] Loos N. (1982). Invasion and Resistance: Aboriginal-European Relations on the North Queensland Frontier 1867-1897.

[bib83] Lourandos H., David B. (1998). Comparing long-term archaeological and environmental trends: north Queensland, arid and semi-arid Australia. The Artefact.

[bib84] Maezumi S.Y., Alves D., Robinson M., de Souza J.G., Levis C., Barnett R.L., de Oliveira E.A., Urrego D., Schaan D., Iriarte J. (2018). The legacy of 4,500 years of polyculture agroforestry in the eastern Amazon. Nat. Plants.

[bib85] Maezumi S.Y., Robinson M., de Souza J., Urrego D.H., Schaan D., Alves D., Iriarte J. (2018). New insights from Pre-Columbian land use and fire management in Amazonian Dark Earth forests. Front. Ecol. Evol..

[bib86] Mandingalbay Yidinji People (2017). http://www.djunbunji.com.au/files/4915/4510/3380/06_WaW_Djunbunji_Dec17_-_Edition_10.pdf.

[bib87] McCracken C.R. (1989). Some Aboriginal walking tracks and camp sites in Douglas Shire, north Queensland. Qld. Archaeolog. Res..

[bib88] McDonald J., Reynen W., Petchey F., Ditchfield K., Byrne C., Vannieuwenhuyse D., Leopold M., Veth P. (2018). Karnatukul (Serpent's Glen): a new chronology for the oldest site in Australia's Western Desert. PLoS One.

[bib89] Meggers B.J. (1971). Amazonia: Man and Culture in a Counterfeit Paradise.

[bib90] Meston A. (1904). Report on Expedition to the Bellenden-Kerr Range. C.A. 36.

[bib91] Metcalfe D.J., Ford A.J., Stork N.E., Turton S. (2008). Floristics and plant diversity of the wet tropics. Living in a Dynamic Tropical Forest Landscape.

[bib92] Metcalfe D.J., Ford A.J. (2009). Re-evaluation of Queensland’s Wet Tropics based on ‘primitive’ plants. Pac. Conserv. Biol..

[bib93] Mjöberg E., Ritchie R. (2015). [1918]. Amongst Stone Age People in the Queensland Wilderness*,* Å Ferrier.

[bib94] Morrill J., Welch D. (2006). [1864]. 17 years wandering among the Aboriginals.

[bib95] Moss P.T., Cosgrove R., Ferrier Å., Haberle S.G., Haberle S.G., David B. (2012). Holocene environments of the sclerophyll woodlands of the Wet Tropics of northeastern Australia. Peopled Landscapes. Archaeological and Biogeographic Approaches to Landscapes.

[bib96] Mulligan J. (1876). Letter to the Editor 3 June 1876.

[bib97] National Environmental Research Program (2014). How would value-adding to indigenous protected areas improve management of wet tropics country? Tropical ecosystems hub, research towards policy brief. http://www.nerptropical.edu.au/sites/default/files/publications/files/3658%20CSIRO%20Policy%203.pdf.

[bib98] National Indigenous Australians Agency (2021). Indigenous protected areas (IPAs). ..

[bib99] Neldner V.J., Niehus R.E., Wilson B.A., McDonald W.J.F., Ford A.J., Accad A. (2019). The Vegetation of Queensland. Descriptions of Broad Vegetation Groups. Version 4.0. https://www.qld.gov.au/environment/plants-animals/plants/ecosystems/broad-vegetation.

[bib100] Neves E.G., Petersen J.B., Bartone R.N., Da Silva C.A., Lehmann J., Kern D.C., Glaser B., Woods W.I. (2003). Historical and socio-cultural origins of Amazonian dark earths. Amazonian Dark Earths: Origin, Properties, Management.

[bib101] Newson L.A. (2009). Conquest and Pestilence in the Early Spanish Philippines.

[bib102] Nunn P.D., Reid N.J.( (2016). Aboriginal memories of inundation of the Australian coast dating from more than 7000 Years ago. Aust. Geogr..

[bib103] O’Leary M., Raders R. (2019). [1918]. The aboriginals parts I-X by coyyan. The Northern Herald, January-May 1918.

[bib104] Pannell S. (2005). Yamani Country: A Spatial History of the Atherton Tableland.

[bib105] Pannell S., Stork N.E., Turton S.M. (2008). Cultural landscapes in the wet tropics. Living in a Dynamic Tropical Forest Landscape.

[bib106] Pascoe B. (2018). Dark Emu: Aboriginal Australia and the Birth of Agriculture.

[bib107] Pedley H. (1992). Aboriginal Life in the Rainforest: By the Aboriginal People of Jumbun.

[bib108] Piperno D.R., Pearsall D.M. (1998). The Origins of Agriculture in the Lowland Neotropics.

[bib109] Queensland Parks, Wildlife Service, Department of Environment and Science (2019). Daintree national Park management plan. https://parks.des.qld.gov.au/__data/assets/pdf_file/0021/168312/daintree-national-park-management-plan-2019.pdf.

[bib110] Rangan H., Bell K.L., Baum D.A., Fowler R., McConvell P., Saunders T., Spronck S., Kull C.A., Murphy D.J. (2015). New genetic and linguistic analyses show ancient human influence on Boabab evolution and distribution in Australia. PLoS One.

[bib111] Review Steering Committee (1998). The Review of Aboriginal Involvement in the Management of the Wet Tropics World Heritage Area: Thematic Presentation of the 14 Terms of Reference or ‘Which Way Our Cultural Survival’. https://www.wettropics.gov.au/site/user-assets/REVIEW.pdf.

[bib112] Ricketts T.H., Soares-Filho B., da Fonseca G.A.B., Nepstrad D., Pfaff A., Petsonk A., Anderson A., Boucher D., Cattaneo A., Conte M. (2010). Indigenous lands, protected areas, and slowing climate change. PLoS Biol..

[bib113] Roberts P. (2019). Tropical Forests in Prehistory, History, and Modernity.

[bib114] Roberts P., Petraglia M.D. (2015). Pleistocene rainforests: barriers or attractive environments for early human foragers?. World Archaeol..

[bib115] Roberts P., Perera N., Wedage O., Deraniyagala S., Perera J., Eregama S., Gledhill A., Petraglia M.D., Lee-Thorp J.A. (2015). Direct evidence for human reliance on rainforest resources in late Pleistocene Sri Lanka. Science.

[bib116] Roberts P., Hunt C., Arroyo-Kalin M., Evans D., Boivin N.( (2017). The deep human prehistory of global tropical forests and its relevance for modern conservation. Nat. Plants..

[bib117] Roberts P., Perera N., Wedage O., Deraniyagala S.U., Perera J., Eregama S., Gledhill A., Petraglia M.D., Lee-Thorp J.A. (2017). Fruits of the forest: human stable isotope ecology and rainforest adaptations in Late Pleistocene and Holocene (*~* 36 to 3 ka) Sri Lanka. J. Hum. Evol..

[bib118] Roberts P., Boivin N., Kaplan J. (2018). Finding the anthropocene in tropical forests. Anthropocene.

[bib119] Rossetto M., Ens E.J., Honings T., Wilson P.D., Yap J.-&.S., Costello O., Round E.R., Bowern C. (2017). From Songlines to genomes: prehistoric assisted migration of a rain forest tree by Australian Aboriginal people. PLoS One.

[bib120] Sanderson R. (2008). Re-writing the history of Australian tropical rainforests: ‘alien invasives’ or ‘ancient indigenes’?. Environ. Hist..

[bib121] Sheil D., Boissière M., Beaudoin G. (2015). Unseen sentinels: local monitory and control in conservation’s blind spots. Ecol. Soc..

[bib122] Shepard J.R.G., Ramirez H. (2011). “Made in Brazil”: human dispersal of the Brazil nut (*Bertholletia excelsa*, Lecythidaceae) in ancient amazonia. Eco. Bot..

[bib123] Smith A. (2007). *Ravenshoe Backtracks*. A Guide to a Selection of Misty Mountains Trails, and Stories from Descendants of the Men and Women Who Marked Them Out.

[bib124] Spengler R.N., Mueller N.G. (2019). Grazing animals drove domestication of grain crops. Nat. Plants..

[bib125] Stanton P., Stanton D., Stott M., Parsons M. (2014). Fire exclusion and the changing landscape of Queensland’s Wet Tropics Bioregion 1. The extent and pattern of transition. Aust. For..

[bib126] Stanton P., Parsons M., Stanton D., Stott M. (2014). Fire exclusion and the changing landscape of Queensland’s Wet Tropics Bioregion 2. The dynamics of transition forests and implications for management. Aust. For..

[bib127] Stocker G.C., Mott J.J., Gill A.M., Groves R.H., Noble I.R. (1981). Fire in the tropical forests and woodlands of northern Australia. Fire and the Australian Biota.

[bib128] Stork N., Goosem S., Turton S.M., Stork N., Turton S. (2008). Australian rainforests in a global context. Living in a Dynamic Tropical Forest Landscape.

[bib129] Summerhayes G.R., Leavesley M., Fairbairn A., Mandui H., Field J., Ford A., Fullagar R. (2010). Human adaptation and plant Use in highland new Guinea 49,000 to 44,000 Years ago. Science.

[bib130] Ter Steege H., Pitman N.C.A., Sabatier D., Baraloto C., Salomao R.O., Guevara J.E., Phillips O.L., Castilho C.V., Magnusson W.E., Molinso J.-F. (2013). Hyperdominance in the Amazonian tree flora. Science.

[bib131] Thomas E., Alcázar Caicedo C., McMichael C.H., Corvera R., Loo J. (2015). Uncovering spatial patterns in the natural and human history of Brazil nut (*Bertholletia excelsa*) across the Amazon Basin. J. Biogeogr..

[bib132] Tracey J.G., Webb L.J. (1975). Vegetation of the Humid Tropical Region of North Queensland.

[bib133] Tracey J.G. (1982). The Vegetation of the Humid Tropical Region of North Queensland.

[bib134] Tuechler A., Ferrier Å., Cosgrove R. (2016). Transforming the inedible to the edible: an analysis of the nutritional returns from Aboriginal nut processing in Queensland’s Wet Tropics. Aust. Archaeol..

[bib135] Turney C.S.M., Kershaw A.P., Moss P., Bird M.I., Fifield L.K., Cresswell R.G., Santos G.M., Di Tada M.L., Hausladen P.A., Zhou Y. (2001). Redating the onset of burning at Lynch’s crater (North Queensland): implications for human settlement in Australia. J. Qua. Sci..

[bib136] UNESCO (2020). Wet tropics of Queensland. https://whc.unesco.org/en/list/486/.

[bib137] Van der Wal J., Shoo L.P., Williams S.E., Ladiges P. (2009). New approaches to understanding late Quaternary climate flcutuations and refugial dynamics in Australia Wet Tropical rain forests. J. Biogeogr..

[bib138] Webb L.J., Tracey J.G., Groves R.H. (1994). The rainforests of northern Australia. Australian Vegetation.

[bib139] Westaway K.E., Louys J., Due Awe R., Morwood M.J., Price G.J., Zhao J.-x., Aubert M., Joannes-Boyau R., Smith T.M., Skinner M.M. (2017). An early modern human presence in Sumatra 73,000-63,000 years ago. Nature.

[bib140] Wet Tropics Aboriginal Cultural and Natural Plan Project Team (2005). Caring for Country and Culture: The Wet Tropics Aboriginal Cultural and Natural Resource Management Plan.

[bib141] Wet Tropics Management Authority (2013). Boundary of the Wet Tropics Bioregion of Queensland. http://www.wettropics.gov.au/site/user-assets/docs/wet-tropics-of-queensland-world-heritage-area.kmz.

[bib142] Wet Tropics Management Authority (2015). State of Wet Tropics Reports 2014-2015: Economic value of the Wet Tropics World Heritage Area.

[bib143] Wet Tropics Management Authority (2016). State of wet tropics report 2015-2016. Ancient, endemic, rare and threatened vertebrates of the wet tropics. https://www.wettropics.gov.au/site/user-assets/docs/sowt2015-16b5-lres.pdf.

[bib144] Wet Tropics Management Authority (2019). Jirrbal traditional Owners return to Urumbal pocket. https://www.wettropics.gov.au/jirrbal-traditional-owners-return-to-urumbal-pocket.

[bib145] Wet Tropics Management Authority (2020). World heritage area facts and figures. https://www.wettropics.gov.au/world-heritage-area-facts-and-figures.html.

[bib146] Wet Tropics Management Authority (2020). Wet tropics management authority. https://www.wettropics.gov.au/wet-tropics-management-authority.

[bib147] Wet Tropics Management Authority (2020). Rainforest aboriginal news. https://www.wettropics.gov.au/rainforest-aboriginal-news.

[bib148] Wet Tropics Management Authority (2020). Cooperative Management Agreements: A Guide for Rainforest Aboriginal People.

[bib149] Wet Tropics Management Authority (2020). Wet tropics strategic plan 2020-2030. https://www.wettropics.gov.au/StrategicPlan.

